# Initial Treatment Patterns and Survival Outcomes of Mantle Cell Lymphoma Patients Managed at Chinese Academic Centers in the Rituximab Era: A Real-World Study

**DOI:** 10.3389/fonc.2021.770988

**Published:** 2022-01-04

**Authors:** Meng Wu, Yun Li, Huiqiang Huang, Wei Xu, Yanyan Wang, Haiwen Huang, Weili Zhao, Shuo Liu, Pengpeng Xu, Zhengming Chen, Jun Zhu, Yuqin Song, Jia Ruan, Depei Wu

**Affiliations:** ^1^ Key Laboratory of Carcinogenesis and Translational Research (Ministry of Education), Department of Lymphoma, Peking University Cancer Hospital & Institute, Beijing, China; ^2^ National Clinical Research Center for Hematologic Diseases, Jiangsu Institute of Hematology, The First Affiliated Hospital of Soochow University, Suzhou, China; ^3^ Collaborative Innovation Center of Hematology, Soochow University, Suzhou, China; ^4^ Department of Medical Oncology, Sun Yat-Sen University Cancer Center, Guangzhou, China; ^5^ Department of Hematology, The First Affiliated Hospital of Nanjing Medical University, Jiangsu Province Hospital, Nanjing, China; ^6^ Shanghai Institute of Hematology, State Key Laboratory of Medical Genomics, National Research Center for Translational Medicine at Shanghai, Ruijin Hospital Affiliated to Shanghai Jiao Tong University School of Medicine, Shanghai, China; ^7^ Weill Cornell Medicine and New York Presbyterian Hospital, New York, NY, United States

**Keywords:** mantle cell lymphoma, initial treatment, real-world data, the rituximab era, multicenter research

## Abstract

**Purpose:**

The aim of the study was to delineate the disease characteristics, the initial treatment patterns, and survival in patients with mantle cell lymphoma (MCL) managed in the real world.

**Methods:**

Data of 518 MCL patients from 5 major Chinese Hematology Centers in the period from 2007 to 2017 were retrospectively analyzed.

**Results:**

The median age was 58 years. Of the patients, 88.6% had Eastern Cooperative Oncology Group Performance Status (ECOG PS) 0–1 and 80.7% had advanced-stage disease. Ki67 expression was <30% in 39.6% of the patients, and 43.2% of patients were categorized into a low-risk group based on the Mantle Cell Lymphoma International Prognostic Index (MIPI) scoring system. Overall, 73.4% of the patients received rituximab as their first-line therapy. The most commonly used chemotherapy was the CHOP-like (cyclophosphamide, hydroxydaunomycin, oncovin, and prednisone) regimen (45.2%), followed by high-dose cytarabine-containing chemotherapy (31.3%) and bendamustine (3.3%). Of the patients, 13.7% (*n* = 71) underwent consolidative autologous stem cell transplantation (ASCT), and 19.3% (*n* = 100) received novel agents containing first-line regimens. With a median follow-up time of 52 months, the 3- and 5-year overall survival (OS) rates were 73.7% and 61.4%, respectively. Age ≤60 years, ECOG PS 0–1, stages I–II, normal lactate dehydrogenase (LDH), absence of bone marrow involvement, Ki67 <30%, and lower-risk IPI/MIPI scores were significantly associated with improved OS (*p* < 0.05). The inclusion of rituximab improved the 5-year OS, with borderline significance (62.5% *vs*. 55.2%, *p* = 0.076). High-dose cytarabine-containing chemotherapy showed significant clinical benefit in 5-year OS (72.1% *vs*. 55.9%, *p* = 0.010). Patients with ASCT had better 5-year OS in the younger (≤60 years) age group (87.2% *vs*. 64.8%, *p* = 0.002).

**Conclusion:**

This large retrospective dataset unequivocally confirmed the survival advantage afforded by cytarabine-containing regimen and ASCT in a first-line setting under real-world management in the rituximab era.

## 1 Introduction

Mantle cell lymphoma (MCL) is an incurable and heterogeneous disease characterized by chromosomal t(11:14)(q13;32) translocation and cyclin D1 overexpression (1). It represents about 6%–9% of all newly diagnosed cases of non-Hodgkin’s lymphomas (NHLs) in Western countries (1, 2). It has been reported that the incidence in Asian countries is lower (about 1.5%–3.4%) (3, 4). Whether the clinical features in the Asian population are the same as those of the Western population is unknown. The clinical outcomes of MCL are associated with disease biology and patient factors. About 10%–20% of MCL patients present with an indolent course and can be managed with the “watch-and-wait” strategy for a period of time (5). In patients with symptomatic and aggressive course, intensive chemotherapy containing high-dose cytarabine followed by consolidative autologous stem cell transplantation (ASCT) have been used in younger and fit patients, while less intensive chemo-immunotherapy and maintenance therapy are suitable for older or physically unfit patients. Many Chinese patients cannot tolerate full-dose intensive chemotherapy or may choose the less intensive chemotherapy as the first-line therapy due to personal preference. There is a paucity of reported data on the initial treatment patterns and clinical outcomes in Chinese patient populations managed in the rituximab era. The aim of the study was to delineate the disease characteristics, treatment strategies, and survival in real-world Chinese MCL patients.

## 2 Methods

### 2.1 Patients and Data Collection

Retrospective data of newly diagnosed MCL patients were collected from 5 major Chinese Hematology Centers in Beijing, Guangzhou, Nanjing, Shanghai, and Suzhou in the period from 2007 to 2017. The diagnosis for MCL was based on characteristic immunophenotype and cyclin D1 immunohistochemistry staining. The stage was evaluated by PET/CT or CT scan of the neck, chest, abdomen, and pelvis and by bone marrow biopsy. For patients with gastrointestinal involvement indicated during imaging examination, gastrointestinal endoscopy was conducted. Patients with unconfirmed diagnostic histology, unknown initial treatment, or missing outcome data were excluded. The analysis was approved by the Institutional Review Board of the lead institution of The First Affiliated Hospital of Soochow University and all other participating institutions. Data cut for the last follow-up occurred on 31 October 2019.

In total, 518 cases were included in the final analysis, out of 605 cases initially diagnosed with MCL. Of the 87 cases that were excluded, 51 cases were subsequently determined by the Pathology Department at each center to be non-MCL in December 2019 based on the 2016 revision of the World Health Organization Classification of lymphoid neoplasms (6). Another 36 cases were excluded due to conflicting survival data between the first and the second follow-up, conducted between July 2018 and October 2019.

### 2.2 Statistical Analysis

Median follow-up time was estimated for overall survival (OS) using the reverse Kaplan–Meier method. OS time was calculated from the date of diagnosis to the date of death or date of the last follow-up, whichever comes first. Differences between the clinical characteristics and therapy strategies were analyzed with the chi-square test. The Kaplan–Meier estimator was used to estimate survival probability. Survival differences between groups were calculated using the log-rank test for statistical significance. The confidence intervals of the survival rates were calculated using Greenwood’s formula. Univariate and multivariate analyses with the Cox proportional hazards model were conducted to estimate the effects of prognostic factors on survival. All statistical tests were two-sided, with an alpha level of 0.05 as the significance cutoff. All analyses were performed in SAS statistical software, version 9.4 (SAS Institute, Cary, NC, USA).

## 3 Results

### 3.1 Patient Characteristics

A total of 518 newly diagnosed MCL patients with a confirmed diagnosis and longitudinal treatment history and outcome data were included in the analysis. The median age was 58 years (range = 28–83 years), with 59.7% of the patients not older than 60 years; the male-to-female ratio was 3.35:1. Of all patients, 88.6% had Eastern Cooperative Oncology Group Performance Status (ECOG PS) 0–1 and 80.7% had advanced-stage disease. Ki67 expression was <30% in 39.6% and was ≥30% in 44.6% of the patients. About 27.6% of the patients presented with International Prognostic Index (IPI) of 3–5, and 37.8% had intermediate- and high-risk Mantle Cell Lymphoma International Prognostic Index (MIPI) scores (**Table 1**).

**Table 1:** Patient characteristics in different age groups.

	Total *N* (%)	Age (years)		*p*-value
		≤60, *N* (%)	>60, *N* (%)	
**No. of patients**	518	309 (59.7)	209 (40.3)	–
**Age**	median (range)	58.00	(28.00–83.00)	–
**Gender**				
Male	399 (77.0)	243 (78.6)	156 (74.6)	0.288
Female	119 (23.0)	66 (21.4)	53 (25.4)	
**ECOG PS**				
0–1	459 (88.6)	284 (91.9)	175 (83.7)	0.003
>1	44 (8.5)	17 (5.5)	27 (12.9)	
Missing	15 (2.9)	8 (2.6)	7 (3.3)	
**Ann Arbor stage**				
I–II	62 (12.0)	34 (11.0)	28 (13.4)	0.480
III–IV	418 (80.7)	249 (80.6)	169 (80.9)	
Missing	38 (7.3)	26 (8.4)	12 (5.7)	
**MIPI**				
High risk	96 (18.5)	24 (7.8)	72 (34.4)	<0.001
Intermediate risk	100 (19.3)	23 (7.4)	77 (36.8)	
Low risk	224 (43.2)	191 (61.8)	33 (15.8)	
Missing	98 (18.9)	71 (23.0)	27 (12.9)	
**IPI**				
0–1	142 (27.4)	129 (41.7)	13 (6.2)	<0.001
2	138 (26.6)	75 (24.3)	63 (30.1)	
3	112 (21.6)	31 (10.0)	81 (38.8)	
4–5	31 (6.0)	2 (0.6)	29 (13.9)	
Missing	95 (18.3)	72 (23.3)	23 (11.0)	
**LDH**				
Elevated	96 (18.5)	49 (15.9)	47 (22.5)	0.043
Normal	220 (42.5)	139 (45.0)	81 (38.8)	
Missing	202 (39.0)	121 (39.2)	81 (38.8)	
**BM positive**				
No	247 (47.7)	165 (53.4)	82 (39.2)	0.002
Yes	217 (41.9)	115 (37.2)	102 (48.8)	
Missing	54 (10.4)	29 (9.4)	25 (12.0)	
**Ki67**				
<30%	205 (39.6)	136 (44.0)	69 (33.0)	0.032
≥30%	231 (44.6)	130 (42.1)	101 (48.3)	
Missing	82 (15.8)	43 (13.9)	39 (18.7)	

ECOG PS, Eastern Cooperative Oncology Group Performance Status; MIPI, Mantle Cell Lymphoma International Prognostic Index; IPI, International Prognostic Index; LDH, lactate dehydrogenase; BM, bone marrow.

The elderly group (patients over 60 years, 40.3%) was similar to the younger group (patients younger than 60 years) in gender distribution and stage status. Patients in the elderly group had a higher rate of poor ECOG PS, Ki67 expression ≥30%, elevated lactate dehydrogenase (LDH), and bone marrow involvement compared with younger patients. Since age was an important factor in both MIPI and IPI scores, the proportions of patients with intermediate- and high-risk MIPI and with IPI scores of 3–5 in the elderly group were higher than that in the younger group (71.2% *vs*. 15.2%, *p* < 0.001; 52.7% *vs*. 10.6%, *p* < 0.001, respectively) (**Table 1**).

### 3.2 Treatment Description

In the entire cohort, 73.4% (*n* = 380) of patients received rituximab as their first-line therapy. The use of rituximab was similar in the different age groups. A total of 162 (31.3%) patients were treated with high-dose cytarabine-containing chemotherapy. The most commonly used cytarabine-containing regimens were Hyper-CVAD (cyclophosphamide, vincristine, adriamycin, and dexamethasone) (83 cases) and DHAP (dexamethasone, Ara C, and cisplatin) (73 cases). The proportion of patients receiving high-dose cytarabine-containing chemotherapy was higher in the younger group than that in the elderly group (42.4% *vs*. 14.8%, *p* < 0.001).

Apart from the high-dose cytarabine-containing chemotherapy regimens, the most commonly used chemotherapy regimen was the CHOP-like (cyclophosphamide, hydroxydaunomycin, oncovin, and prednisone) regimen (45.2%). The proportions of patients using other regimens, such as bendamustine-based, CVP-like (cyclophosphamide, vincristine, and prednisolone), GemOx (gemcitabine and oxaliplatin), and FC (fludarabine and cyclophosphamide), were all less than 5%.

A total of 71 patients (13.7%) received ASCT as consolidation therapy. In the younger group, 68 patients (22.0%) received ASCT as consolidation therapy. Three patients over 60 years received ASCT, including a 68-year-old patient.

Among the 518 patients, 100 (19.3%) received regimens containing new drugs as their first-line therapy, such as bortezomib (*n* = 28, 5.4%), ibrutinib (*n* = 20, 3.9%), lenalidomide (*n* = 18, 3.5%), and thalidomide (*n* = 34, 6.6%) (**Table 2**).

**Table 2:** Treatment summary in different age groups.

	Total *N* (%)	Age (years)		*p*-value
		≤60, *N* (%)	>60, *N* (%)	
**No. of patients**	518	309 (59.7%)	209 (40.3%)	–
**Rituximab-containing regimen**				
No	109 (21.0)	65 (21.0)	44 (21.1)	0.945
Yes	380 (73.4)	228 (73.8)	152 (72.7)	
Missing	29 (5.6)	16 (5.2)	13 (6.2)	
**HDAC regimen**				
No	326 (62.9)	161 (52.1)	165 (78.9)	<0.001
Yes	162 (31.3)	131 (42.4)	31 (14.8)	
Missing	30 (5.8)	17 (5.5)	13 (6.2)	
**ASCT**				
No	419 (80.9)	225 (72.8)	194 (92.8)	<0.001
Yes	71 (13.7)	68 (22.0)	3 (1.4)	
Missing	28 (5.4)	16 (5.2)	12 (5.7)	
**First-line chemotherapy**				
CHOP-like	234 (45.2)	140 (45.3)	94 (45.0)	<0.001
Hyper-CVAD	83 (16.0)	62 (20.1)	21 (10.0)	
DHAP	73 (14.1)	62 (20.1)	11 (5.3)	
V-CAP	18 (3.5)	6 (1.9)	12 (5.7)	
Bendamustine-based	17 (3.3)	1 (0.3)	16 (7.7)	
CVP-like	12 (2.3)	4 (1.3)	8 (3.8)	
GemOx	12 (2.3)	0 (0.0)	12 (5.7)	
FC	9 (1.7)	5 (1.6)	4 (1.9)	
Others	23 (4.4)	10 (3.2)	13 (6.2)	
Missing	37 (7.1)	19 (6.1)	18 (8.6)	
**First-line novel agents**				
Bortezomib	28 (5.4)	14 (4.5)	14 (6.7)	<0.001
Ibrutinib	20 (3.9)	4 (1.3)	16 (7.7)	
Lenalidomide	18 (3.5)	9 (2.9)	9 (4.3)	
Thalidomide	34 (6.6)	14 (4.5)	20 (9.6)	
No	418 (80.7)	268 (86.7)	150 (71.8)	

HDAC, high-dose cytarabine-containing; ASCT, autologous stem cell transplantation; CHOP, cyclophosphamide, hydroxydaunomycin, oncovin, and prednisone; CVAD, cyclophosphamide, vincristine, adriamycin, and dexamethasone; DHAP, dexamethasone, Ara C, and cisplatin; V-CAP, bortezomib, cyclophosphamide, adriamycin, and prednisone; CVP, cyclophosphamide, vincristine, and prednisolone; GemOx, gemcitabine and oxaliplatin; FC, fludarabine and cyclophosphamide.

### 3.3 Comparison of Survival Between Different Patient Characteristics

**Figure 1:**
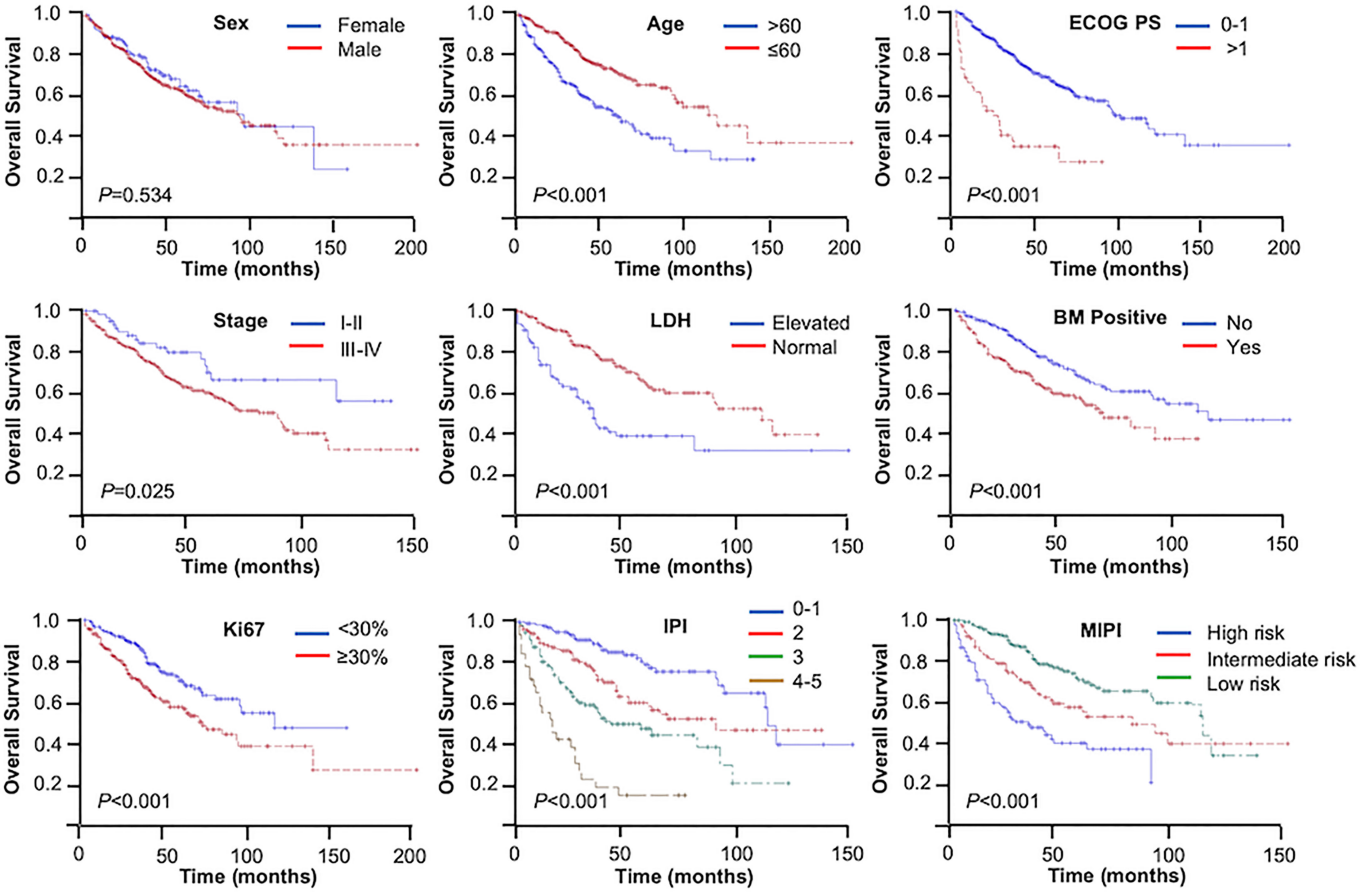
Kaplan–Meier curves: overall survival (OS) in different patient characteristics.

The median follow-up time was 52 months. During the analysis, 188 (36.3%) patients had died. The 3- and 5-year OS rates were 73.7% and 61.4%, respectively. Based on log-rank test analysis, the median OS for patients in the younger group was 119 months compared with 59 months in the elderly group (3-year OS = 81.3% *vs*. 62.8%, 5-year OS = 70.7% *vs*. 48.2%, *p* < 0.001). Except for gender, the stratification of all other baseline characteristics (including ECOG PS, Ann Arbor stage, LDH, bone marrow status, Ki67, IPI, and MIPI) provided a reliable prediction of the OS rates (*p* < 0.001, *p* = 0.025, *p* < 0.001, *p* < 0.001, *p* < 0.001, *p* < 0.001, and *p* < 0.001, respectively) (**Figure 1**).

### 3.4 Comparison of Individual Treatment Regimens

#### 3.4.1 Rituximab

**Figure 2:**
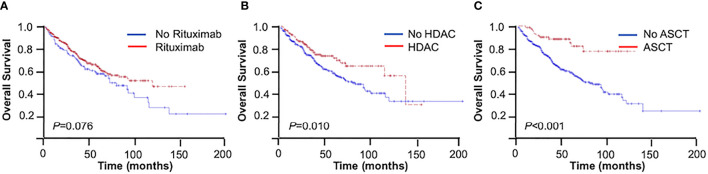
Overall survival (OS) of mantle cell lymphoma (MCL) patients. **(A)** With or without rituximab. **(B)** With or without high-dose cytarabine-containing (HDAC) regimen. **(C)** With or without autologous stem cell transplantation (ASCT).

Patients receiving rituximab trended toward better outcomes compared with those without rituximab in the entire cohort (3-year OS = 74.4% *vs*. 69.0%, 5-year OS = 62.5% *vs*. 55.2%, *p* = 0.076) (**Figure 2A**). The inclusion of rituximab had similar efficacy in both the younger group and the elderly group (younger group: 3-year OS = 81.0% *vs*. 79.0%, 5-year OS = 72.7% *vs*. 64.9%, *p* = 0.169; elderly group: 3-year OS = 64.6% *vs*. 53.5%, 5-year OS = 48.6% *vs*. 39.9%, *p* = 0.142).

#### 3.4.2 First-Line Chemotherapy

The use of high-dose cytarabine showed significant clinical benefit in OS (3-year OS = 77.2% *vs*. 71.1%, 5-year OS = 72.1% *vs*. 55.9%, *p* = 0.010) (**Figure 2B**). Nearly half of the patients (45.2%) received CHOP-like regimens, with the 3- and 5-year OS at 73.9% and 58.0%, respectively. In addition, patients receiving moderate regimens, such as CVP-like regimen, had a 3-year OS of 50.0%. Among the patients who received bendamustine-based regimens (*n* = 17, 3.3%), the 3- and 5-year OS rates were 88.2% and 69.1%, respectively. The 3- and 5-year OS rates were 81.4% and 74.0% in patients receiving the V-CAP (bortezomib, cyclophosphamide, adriamycin, and prednisone) regimen (*n* = 18, 3.5%) (**Table 3**).

**Table 3:** Overall survival (OS) stratified by clinical features and therapy strategies in mantle cell lymphoma (MCL).

	Total (event)	Median (95% CI)	3-year OS (95% CI)	5-year OS (95% CI)	*p*-value
**Overall**	514 (188)	95.0 (73.0–119.0)	73.7 (69.7–77.8)	61.4 (56.4–66.4)	
**Gender**					
Male	399 (149)	95.0 (69.0–119.0)	72.6 (67.9–77.3)	60.9 (55.3–66.6)	0.534
Female	119 (40)	95.0 (64.0–)	77.4 (69.4–85.5)	62.8 (52.2–73.4)	
**Age (years)**					
≤60	309 (87)	119.0 (96.0–)	81.3 (76.5–86.0)	70.7 (64.6–76.8)	<0.001
>60	209 (102)	59.0 (42.0–73.0)	62.8 (55.9–69.7)	48.2 (40.3–56.1)	
**ECOG PS**					
0–1	459 (154)	97.0 (76.0–124.0)	77.5 (73.4–81.6)	64.4 (59.1–69.7)	<0.001
>1	44 (30)	19.0 (5.0–35.0)	34.1 (19.6–48.6)	31.1 (16.8–45.5)	
**Ann Arbor stage**					
I–II	62 (16)	(62.0–)	81.2 (70.6–91.8)	71.9 (58.2–85.6)	0.025
III–IV	418 (166)	74.0 (64.0–97.0)	71.6 (67.0–76.2)	58.1 (52.5–63.6)	
**MIPI**					
High risk	96 (55)	28.0 (18.0–47.0)	47.5 (37.0–58.0)	36.5 (25.3–47.8)	<0.001
Intermediate risk	100 (43)	86.0 (42.0–)	67.4 (57.7–77.1)	54.8 (43.6–66.0)	
Low risk	224 (60)	120.0 (95.0–)	83.5 (78.2–88.8)	69.7 (62.1–77.3)	
**IPI**					
0–1	142 (30)	120.0 (99.0–)	89.8 (84.5–95.1)	80.0 (71.9–88.1)	<0.001
2	138 (47)	95.0 (53.0–)	76.5 (68.7–84.3)	58.8 (48.4–69.2)	
3	112 (56)	42.0 (29.0–97.0)	57.8 (48.2–67.4)	45.7 (34.9–56.5)	
4–5	31 (25)	16.0 (7.0–27.0)	20.3 (4.9–35.6)	12.2 (0.0–24.8)	
**LDH**					
Elevated	96 (51)	37.0 (26.0–48.0)	50.7 (39.8–61.6)	36.7 (25.2–48.2)	<0.001
Normal	220 (67)	119.0 (72.0–)	81.4 (75.8–86.9)	65.9 (58.0–73.7)	
**BM positive**					
No	247 (74)	119.0 (76.0–)	80.2 (74.7–85.7)	66.5 (59.1–73.8)	<0.001
Yes	217 (92)	69.0 (48.0–98.0)	66.9 (60.4–73.4)	54.7 (47.0–62.4)	
**Ki67**					
<30%	205 (56)	119.0 (82.0–)	84.9 (79.6–90.2)	69.1 (61.2–76.9)	<0.001
≥30%	231 (94)	71.0 (53.0–95.0)	68.3 (61.8–74.7)	55.7 (48.1–63.4)	
**Rituximab-containing regimen**					
No	109 (54)	74.0 (46.0–103.0)	69.0 (60.0–78.0)	55.2 (44.9–65.5)	0.076
Yes	380 (128)	97.0 (69.0–)	74.4 (69.7–79.1)	62.5 (56.6–68.3)	
**HDAC regimen**					
No	326 (137)	74.0 (59.0–96.0)	71.1 (65.9–76.3)	55.9 (49.7–62.2)	0.010
Yes	162 (44)	143.0 (119.0–)	77.2 (70.2–84.1)	72.1 (64.3–79.9)	
**First-line chemotherapy**					
CHOP-like	234 (93)	73.0 (62.0–143.0)	73.9 (67.9–79.9)	58.0 (50.7–65.3)	<0.001
Hyper-CVAD	83 (31)	119.0 (60.0–)	68.8 (58.4–79.3)	63.6 (52.4–74.9)	
DHAP	73 (9)	(74.0–)	85.8 (76.3–95.3)	85.8 (76.3–95.3)	
V-CAP	18 (5)	(37.0–)	81.4 (62.2–100.0)	74.0 (51.7–96.2)	
**Bendamustine-based**	17 (5)	(47.0–)	88.2 (72.9–100.0)	69.1 (46.4–91.7)	
CVP-like	12 (8)	40.5 (5.0–57.0)	50.0 (21.7–78.3)	–	
GemOx	12 (5)	42.0 (1.0–)	65.6 (38.1–93.1)	43.8 (4.2–83.3)	
FC	9 (8)	26.0 (3.0–103.0)	44.4 (12.0–76.9)	22.2 (0.0–49.4)	
**ASCT**					
No	419 (173)	73.0 (62.0–96.0)	70.6 (65.9–75.2)	56.3 (50.7–62.0)	<0.001
Yes	71 (11)	(–)	87.8 (79.8–95.8)	84.5 (74.5–94.4)	

ECOG PS, Eastern Cooperative Oncology Group Performance Status; MIPI, Mantle Cell Lymphoma International Prognostic Index; IPI, International Prognostic Index; LDH, lactate dehydrogenase; BM, bone marrow; HDAC, high-dose cytarabine-containing; ASCT, autologous stem cell transplantation; CI, confidence interval; CHOP, cyclophosphamide, hydroxydaunomycin, oncovin, and prednisone; CVAD, cyclophosphamide, vincristine, adriamycin, and dexamethasone; DHAP, dexamethasone, Ara C, and cisplatin; V-CAP, bortezomib, cyclophosphamide, adriamycin, and prednisone; CVP, cyclophosphamide, vincristine, and prednisolone; GemOx, gemcitabine and oxaliplatin; FC, fludarabine and cyclophosphamide.

#### 3.4.3 Autologous Stem Cell Transplant

In the entire cohort, patients with consolidative ASCT achieved 3- and 5-year OS rates of 87.8% and 84.5%, respectively, which were significantly higher than those in patients without ASCT (3-year OS = 70.6%, 5-year OS = 56.3%, *p* < 0.001) (**Figure 2C**). In the younger group, patients with consolidative ASCT had better survival outcomes (3-year OS = 87.2% *vs*. 78.4%, 5-year OS = 87.2% *vs*. 64.8%, *p* = 0.002).

### 3.5 Prognostic Factors

The results of the univariate analysis of independent factors are listed in **Table 4**. Based on the multivariate analysis, high-risk MIPI [hazard ratio (HR) = 2.32, 95% CI = 1.30–4.14, *p* = 0.0042), Ki67 ≥30% (HR = 2.11, 95% CI = 1.37–3.24, *p* = 0.0007), treatment with a rituximab-containing regimen (HR = 0.55, 95% CI = 0.33–0.91, *p* = 0.0197), and treatment with a high-dose cytarabine-containing regimen (HR = 0.50, 95% CI = 0.27–0.92, *p* = 0.0252) were independent risk factors for the prediction of OS.

**Table 4:** Univariate and multivariate predictor analyses.

	Univariable Cox		Multivariable Cox	
Variable	HR (95%CI)	*p*-value	HR (95%CI)	*p*-value
Ann Arbor stages III–IV	3.26 (1.32–8.04)	0.0104	2.22 (0.88–5.63)	0.0914
BM positive	1.86 (1.25–2.78)	0.0022	1.49 (0.96–2.32)	0.0749
MIPI				
High risk	4.18 (2.49–6.99)	<0.0001	2.32 (1.30–4.14)	0.0042
Intermediate risk	1.73 (1.10–2.72)	0.0183	1.31 (0.79–2.15)	0.2937
Ki67 ≥30%	2.32 (1.52–3.54)	<0.0001	2.11 (1.37–3.24)	0.0007
Rituximab regimen	0.62 (0.38–1.00)	0.0505	0.55 (0.33–0.91)	0.0197
HDAC regimen	0.34 (0.20–0.61)	0.0002	0.50 (0.27–0.92)	0.0252
ASCT	0.39 (0.19–0.80)	0.0102	0.65 (0.30–1.42)	0.2858

ECOG PS, Eastern Cooperative Oncology Group Performance Status; LDH, lactate dehydrogenase; BM, bone marrow; MIPI, Mantle Cell Lymphoma International Prognostic Index; IPI, International Prognostic Index; HDAC, high-dose cytarabine-containing; ASCT, autologous stem cell transplantation; CI, confidence interval.

## 4 Discussion

This retrospective, multicenter, real-world study presents the largest Chinese patient cohort of MCL to date. MCL is a heterogeneous and incurable type of lymphoma with highly varied clinical courses. The treatment strategies for MCL have evolved rapidly during the last decade, while the implementation of treatment guidelines remains variable globally due to local practices and access to care. In our study, we investigated the association between the clinical features, initial treatment strategies, and outcomes of MCL patients seeking initial treatment at major academic hematology centers within China.

The 3- and 5-year OS rates of the entire cohort were 73.7% and 61.4% in this study. In the younger group, the 3- and 5-year OS rates were 81.3% and 70.7%, respectively. In contrast, these were 62.8% and 48.2% in the elderly group, respectively. The survival rate appeared to be higher than other real-world data reported from the UK (3-year OS = 43.9%) (7) or Scandinavia (3-year OS = 51%–61%) (8). Several factors potentially account for the differences. Patients with complete diagnosis and treatment data available in this study had a median age of 58 years, which was younger than the median age of patients in Western populations (9). The majority of patients seeking care at major medical centers had good performance status, and nearly half of the patients had low-risk MIPI/IPI scores, which correlated with favorable survival. In comparison to population-based studies, the improved survival from the current study may reflect referral and treatment decisions that were selected for younger and fit patients at the major urban referral centers. Nonetheless, the clinical parameters retained robust prognostic significance within the study cohort.

Combining rituximab in the CHOP regimen had been confirmed to improve the objective response rate and the complete remission rate compared to chemotherapy alone in a prospective randomized trial in young MCL patients in 2005 (10). Rituximab chemotherapy has since been incorporated into the National Comprehensive Cancer Network (NCCN) guideline. The recommended frontline treatment for younger patients was intensive chemotherapy with a high-dose cytarabine-containing regimen with rituximab. The survival benefit of adding rituximab to the CHOP regimen has also been demonstrated in previous randomized (11) and observational (12) studies for elderly MCL patients. The bendamustine plus rituximab regimen conferred better progression-free survival (PFS) in elderly patients compared with the R-CHOP regimen in the StiL and BRIGHT studies (13, 14). Since the expense of rituximab could not be covered by government-sponsored medical insurance until September 2017, a number of patients enrolled before 2017 opted not to use rituximab for economic reasons. In addition, China has been an endemic area for hepatitis B virus (HBV) infection. Thus, some patients had a high level of HBV DNA and could not receive rituximab treatment. Consequently, only 73.4% of patients received rituximab as part of their initial therapy in our study. Compared to those without rituximab, patients receiving rituximab in our study had superior OS with borderline significance (3-year OS = 74.4% *vs*. 69.0%, 5-year OS = 62.5% *vs*. 55.2%, *p* = 0.076). The benefit of rituximab on survival was consistent with the results of other real-world studies (7, 8). Our data supported that immunochemotherapy was the cornerstone of MCL induction in the rituximab era. The survival data with rituximab maintenance after induction therapy or consolidative ASCT were not collected in this analysis; therefore, their impact on PFS and OS in MCL Chinese patients is undefined (15, 16).

Apart from rituximab, intensive chemotherapy and cytarabine-containing regimens were the most important factors in induction therapy for younger MCL patients, as demonstrated in regimens such as R-DHAP or R-DHAx (rituximab, dexamethasone, cytarabine, and oxaliplatin) alternating with R-CHOP, R-Hyper-CVAD, or Nordic MCL studies (15, 17–22). Our study demonstrated the benefits of high-dose cytarabine-containing regimens in MCL patients (3-year OS = 77.2% *vs*. 71.1%, 5-year OS = 72.1% *vs*. 55.9%, *p* = 0.010). Data on the efficacy of and tolerance to cytarabine-containing regimens of elderly Chinese patients remain scanty and deserve attention in future studies.

The survival benefit of ASCT in MCL has been well demonstrated (23, 24). In our study, consolidative ASCT was significantly associated with improved OS in the younger group (3-year OS = 87.2% *vs*. 78.4%, 5-year OS = 87.2% *vs*. 64.8%, *p* = 0.002). However, less than one-third of the young MCL patient group underwent ASCT in the real world (22.0% from our dataset and 17%–31% from other real-world studies) (7, 8, 25). Several factors may have limited broad access to ASCT, including: 1) comorbidities contraindicating ASCT; 2) failure in achieving remission; 3) unsuccessful harvest of stem cells; 4) patient preference due to treatment costs; and 5) availability of local expertise for ASCT.

Novel agents have made their way into real-world practice, first through the introduction of global clinical trials, followed by market access for approved new drugs in relapsed and refractory diseases. Bortezomib, lenalidomide, bendamustine, and Bruton’s tyrosine kinase (BTK) inhibitors have all been approved for the treatment of relapsed and refractory MCL. In recent years, VR-CAP (bortezomib, rituximab, cyclophosphamide, adriamycin, and prednisone) and lenalidomide + rituximab regimens have shown favorable survival outcomes when compared with the R-CHOP regimen directly or with historical data (26–28). Compared with other drugs, BTK inhibitors as single agents have shown higher overall response rates, complete remission rates, and PFS, contributing to improved survival of relapsed and refractory MCL patients (29, 30). The survival benefits conferred by BTK inhibitors in Chinese patients have also been reported (31). In addition, BTK inhibitors are being incorporated into first-line regimens in multiple global phase 3 studies. In our study, 100 patients (19.3%) received regimens containing new drugs, namely, VR-CAP containing bortezomib, bendamustine and rituximab (BR) with ibrutinib, and CHOP or GemOx with either lenalidomide or thalidomide, in the context of either global trials or off-label uses. With accelerated new drug approvals for market access in China, Chinese oncologists will be equipped with an increasing number of treatment strategies to benefit patients.

There were several limitations of our study, mostly pertaining to the retrospective nature of a hospital-based cohort. Firstly, our study did not include temporal data of the earliest diagnosis and the commencement of first-line therapy before hospitalization; therefore, the data did not contain information on the “watch-and-wait” strategy. Secondly, the lack of a prior standardization in the induction regimen and drug dosage among the different centers resulted in heterogeneity in first-line treatment. Thirdly, the limited number of patients who received novel agents was suboptimal for a thorough evaluation of their therapeutic benefits.

In conclusion, this large retrospective dataset of MCL patients who received contemporaneous real-world management in Chinese hematology centers confirmed the survival advantage afforded by high-dose cytarabine-containing chemotherapy and consolidative autologous stem cell transplantation in a first-line setting. The majority of patients in this series were not older than 60 years with good performance status and lower-risk MIPI/IPI scores, which correlated with more favorable survival in response to chemo-immunotherapy. The incorporation of novel agents signals infrastructural readiness to explore novel agents and combinations in both first-line and relapsed settings. It remains to be seen whether a broader demographic population of MCL patients would be attracted to the Chinese academic centers for longitudinal follow-up in the novel agent era, including those more elderly and with comorbidities who are unfit for chemotherapy and those asymptomatic who can be initially managed with expectant watch-and-wait.

## Data Availability Statement

The raw data supporting the conclusions of this article will be made available by the authors, without undue reservation.

## Ethics Statement

The studies involving human participants were reviewed and approved by the Institutional Review Board of the lead institution of The First Affiliated Hospital of Soochow University and all other participating institutions. Written informed consent for participation was not required for this study, in accordance with the national legislation and the institutional requirements.

## Author Contributions

MW collected the data and drafted and revised the paper. YL prepared the data and drafted the paper. HuH, WX, HaH, WZ, JZ, and DW participated in designing and revising the paper. SL, YW, and PX collected the data. ZC performed statistical analyses. JR and YS designed the study, interpreted the results, and revised the paper. DW provided logistic support for the study. All authors provided critical comments on the manuscript. All authors contributed to the article and approved the submitted version.

## Funding

This work was supported by grants from National Natural Science Foundation of China (81730003, 81828001, 81972807, and 81970179), National Science and Technology Major Project (2017ZX09304021), National Science and Technology Major Project for “Significant New Drug Development” (2020zx09201-023), National Key R&D Program of China (2019YFC0840604 and 2017YFA0104502), Key R&D Program of Jiangsu Province (BE2019798), Priority Academic Program Development of Jiangsu Higher Education Institutions (PAPD), Jiangsu Medical Outstanding Talents Project (JCRCA2016002), Jiangsu Provincial Key Medical Center (YXZXA2016002), Suzhou Science and Technology Program Project (SLT201911), the Translational Research Grant of NCRCH (no. 2020ZKMB02), and Suzhou Science and Technology Development Project (no. SYS2019039).

## Conflict of Interest

The authors declare that the research was conducted in the absence of any commercial or financial relationships that could be construed as a potential conflict of interest.

## Publisher’s Note

All claims expressed in this article are solely those of the authors and do not necessarily represent those of their affiliated organizations, or those of the publisher, the editors and the reviewers. Any product that may be evaluated in this article, or claim that may be made by its manufacturer, is not guaranteed or endorsed by the publisher.
